# Correlation analysis of circulating tumor cells and Claudin-4 in breast cancer

**DOI:** 10.3389/pore.2023.1611224

**Published:** 2023-07-03

**Authors:** Jie Chai, Xiangli Liu, Xinju Hu, Chunfang Wang

**Affiliations:** ^1^ Pathology Department, The First Affiliated Hospital of Henan University of Traditional Chinese Medicine, Zhengzhou, China; ^2^ Breast Surgery, The First Affiliated Hospital of Henan University of Traditional Chinese Medicine, Zhengzhou, China

**Keywords:** breast cancer, circulating tumor cells, tight junction protein, claudin-4, molecular subtype

## Abstract

**Objective:** We aimed to explore the relationship between peripheral blood circulating tumor cells (CTCs) and the expression of Claudin-4 in patients with breast cancer, and further explore the potential impact on clinical prognosis and risk assessment.

**Methods:** We classified and enumerated circulating tumor cells in the blood of breast cancer patients by CTC-enriched *in situ* hybridization and the detection of Claudin-4 expression by immunohistochemistry. We carried out an analysis of the correlation between the two and the comparison of their impact on clinical parameters and prognosis.

**Results:** There were 38 patients with a low expression of Claudin-4 and 27 patients with a high expression of Claudin-4. Compared with Claudin-4 low-expression patients, the number of CTCs was higher in patients with high Claudin-4 expression (11.7 vs. 7.4, *p* < 0.001). High Claudin-4 expression was associated with a lower count of epithelial CTCs (E-CTCs) (3.4 vs. 5.0, *p* = 0.033), higher counts of mesenchymal CTCs (M-CTC) (4.4 vs. 1.1, *p* < 0.001), and epithelial/mesenchymal CTCs (E/M-CTCs) (4.0 vs. 3.5, *p* = 0.021). The intensity of Claudin-4 was positively correlated with CTC (r_s_ = 0.43, *p* = 0.001). Multivariate COX regression analysis showed that CTC counts (HR = 1.3, *p* < 0.001), Claudin-4 (HR = 4.6, *p* = 0.008), and Lymphatic metastasis (HR = 12.9, *p* = 0.001) were independent factors for poor prognosis. COX regression of CTC classification showed that epithelial/mesenchymal CTCs (E/M-CTC) (HR = 1.9, *p* = 0.001) and mesenchymal CTCs (M-CTC) (HR = 1.5, *p* = 0.001) were independent influencing factors of adverse reactions in breast cancer patients.

**Conclusion:** The number of CTC in breast cancer is positively correlated with the expression of Claudin-4. High CTC counts and a high proportion of M-CTCs correlated with Claudin-4 expression. CTC counts and Claudin-4 expression were independent predictors of poor prognosis in breast cancer patients.

## Introduction

Breast cancer is the most common malignant tumor in women [1, 2]. In 2020, there were an estimated 2,261,419 new breast cancer cases, accounting for 11.7% of the total cancer incidence, surpassing lung cancer and becoming the leading cause of cancer death among women [2]. Early (or operable) and non-inflammatory locally advanced inoperable breast cancers are considered potentially curable, but the prognosis is better with lower stages [3–6], emphasizing the need for screening and early detection [7, 8].

Several countries have breast cancer screening programs based on mammography, and these programs are associated with a 15%–30% decrease in breast cancer-related mortality but not in overall mortality [8–10]. Breast cancer screening programs are mainly based on mammography, and their sensitivity and specificity remain relatively low, leading to several false-positive and false-negative results [8–11]. Therefore, it is particularly important to find highly sensitive indicators of prognosis and recurrent risks of breast cancer [12].

A small amount of circulating tumor cells (CTCs) in peripheral blood can escape the tumor after the epithelial-mesenchymal transition [13]. In addition, monitoring peripheral blood CTCs could fill the diagnostic gap in tumors <5 mm [14]. The monitoring of CTCs is convenient and conducive to the early diagnosis and prognosis evaluation of breast cancer [15, 16]. They act as seeds for metastases and can be classified as epithelial type (E-CTC), mesenchymal type (M-CTC), or intermediate (E/M-CTC) with a transition from epithelial to mesenchymal phenotype. These subtypes can be distinguished based on the expression of surface markers. Claudin-4 is a tight junction protein family member and mainly regulates tight junctions [17]. Therefore, Claudin-4 plays an important role in the occurrence and metastasis of cancer cells [18]. Studies have shown that Claudin-4 is highly expressed in various malignant tumors [19], suggesting that Claudin-4 may become a tumor marker and an emerging target for treatment [20–22]. Claudin-4 has been reported to regulate EMT through p21-activated kinase 4 (PAK4) expression in human breast cancer cells [23]. However, the relationship between CTCs and Claudin-4 in breast cancer is unclear.

Therefore, we speculate that Claudin-4 may be involved in the EMT process by altering CTC numbers and typing. This study aimed to explore the relationship between peripheral blood CTCs and the expression of Claudin-4 in patients with breast cancer and their relationship with the survival and prognosis of breast cancer patients.

## Methods

### Study design and patients

This cross-sectional study included patients who underwent routine surgical resection and were pathologically confirmed as having breast cancer at the First Affiliated Hospital of Henan University of Traditional Chinese Medicine between January 2018 and December 2020. This study was approved by the ethics committee of the First Affiliated Hospital of Henan University of Traditional Chinese Medicine. The requirement for informed consent was waived due to the retrospective nature of the study. The inclusion criteria were: 1) available clinical data, 2) diagnosis of breast cancer and available tissue specimens, and 3) available peripheral blood samples. The exclusion criteria were: 1) incomplete clinical data or 2) recipient of neoadjuvant treatments.

### Data collection

The demographic and clinical data of the patients were collected, including age, sex, lymph node metastasis, carcinogenic molecular detection, pathological type, tumor size, and Ki-67 expression. Molecular subtypes included Luminal A, Luminal B, human epidermal growth factor receptor-2 (HER-2) amplification, and basal-like. Luminal A type showed estrogen-receptor (ER) positive or progesterone-receptor (PR) positive and PR high expression (≥20%), HER2 negative, or Ki-67 low expression (<14%). Luminal (luminal or hormone receptor-positive) type B was divided into two subtypes, Luminal B (HER-2 negative) and Luminal B (HER-2 positive): Luminal B (HER-2 negative): ER and/or PR positive, HER-2 negative, and high expression of Ki-67 (greater than or equal to 14%). Luminal B (HER-2 positive): ER and/or PR positive, HER- 2 overexpression or proliferation, and any level of Ki-67. HER-2 amplification type, also known as HER2 positive (non-Luminal) type, showed ER and PR deletion, HER-2 overexpression or proliferation, and any level of Ki-67. Basal-like breast cancers have low expressions of ER, PR, and HER2 and high expressions of markers of breast basal or myoepithelial cells, such as caveolin-1, p63.

### CTC detection

Peripheral venous blood (5 mL) was collected from an antecubital vein using a vacuum EDTA anticoagulant tube and transferred to a sample storage tube. The tube was inverted >10 times and incubated at 15°C–30°C for 30 min to lyse the cells. The blood was centrifuged at 1850 rpm for 5 min. The supernatant was discarded, and 4 mL of PBS and 1 mL of RI fixative solution were added, vortexed, and let to stand for 8 min at room temperature. The liquid was poured into the filter connected to a vacuum pump with a negative pressure of −0.06 MPa. The fixative (1 mL) was added for 1 h. The membrane was dehydrated with gradient alcohol to capture the cells. Labels for EP-CAM, CK8, and CK8/18 (as epithelial markers) and Vimentin (as mesenchymal markers) produced by CanPatrol Biotechnology Co., Ltd. were used for mRNA fluorescence *in situ* hybridization. Fluorescence from any of the epithelial/mesenchymal makers are indicative of epithelial/mesenchymal-type circulating tumor cells. The membrane was scanned, and the count of each type of cell was analyzed using Isis5 and Metafer3 fluorescence analysis software produced by German Midas. Red fluorescence represented epithelial-type CTCs (E-CTC), green fluorescence represented mesenchymal-type CTCs (M-CTC), and both red and green represented mixed-type CTCs (E/M-CTC).

### Immunohistochemistry

The postoperative specimens were fixed in 10% formalin, dehydrated, paraffin-embedded, and serially cut into 4-μm sections. After dewaxing and rehydrating, the sections were put into ethylenediaminetetraacetic acid (EDTA) solution (pH 8.0) produced by Beijing Soleibao Technology Co., Ltd. at 100°C for 20 min. The endogenous peroxidase activity was blocked with 3% H_2_O_2_ for 10 min. After rinsing with phosphate buffer solution (PBS), the sections were incubated with an anti-rabbit primary antibody for Claudin-4 (Shanghai Jiehao Biotechnology Technology Co., Ltd., CRM-1251) at 37°C for 1 h. After washing with PBS, the sections were incubated with the secondary antibody (Roche Shanghai Co., Ltd., J19565) and rinsed**.** The sections were revealed with diaminobenzidine (DAB), stained with hematoxylin, dehydrated, and mounted. The cells were counted in 10 randomly selected visual fields at ×400 magnification. For number scoring, <5% of positive cells were scored zero, 5%–25% was one, 26%–50% was two, and >50% was three. For intensity scoring, no staining was zero, light yellow was one, brown was two, and tan was three. The number and intensity scores were multiplied; zero points were negative, one to two represented 1+, three to four represented 2+, and five to six represented 3+. Claudin-4 negative and Claudin-4 1+ were considered low expressions. Claudin-4 2+ and 3+ were considered high expressions. The scores were determined by two pathologists with 10 years of experience, and the average value was taken as the final result.

### Follow-up

The patients were followed up every 2 months by telephone contact after enrollment. Patients’ vital signs and disease progression were recorded. If necessary, the patients were admitted to the hospital to evaluate their progress. The expected follow-up time was 24 months. The follow-up endpoint was all-cause death. The follow-up deadline was 30 October 2020.

### Statistical analysis

All data were analyzed using SPSS 20.0 (IBM, Armonk, NY, United States). Continuous data were expressed as means ± standard deviation and analyzed using Student’s t-test. Categorical data were presented as *n* (%) and analyzed using the chi-square test. The relationship between CTCs and Claudin-4 expression was analyzed using Spearman rank correlation and the Kaplan-Meier method was used to draw the survival curve. Log-rank test was used to compare the differences. A Multivariate COX risk regression model was established to analyze the independent adverse factors of prognosis. The statistical analysis of the data from two groups was performed using a *t*-test. The comparisons of multiple groups were performed by one-way ANOVA and then an LSD-t test. *p* < 0.05 was considered to be significant. Two-sided *p*-values < 0.05 were considered statistically significant.

## Results

### Characteristics of the patients

Sixty-five breast cancer patients, 39 to 61 (median, 49) years of age, were included. All patients were women, including 51 with invasive carcinoma and 14 cases of ductal carcinoma *in situ*. There were 48 patients with a tumor <5 cm and 17 with a tumor >5 cm. There were 21 patients with lymph node metastasis and 44 without. There were 12 patients with Ki- 67 < 14% and 53 with Ki-67 ≥ 14% (Table 1).

**TABLE 1:** Characteristics of the patients and CTC counts.

Pathological parameters	Number of cases (%) *n* = 65
Age (years)	
<50	34 (52.3)
≥50	31 (47.7)
Lymph node metastasis	
No	44 (67.7)
Yes	21 (32.3)
Molecular subtype	
Luminal A	7 (10.8)
Luminal B	42 (64.6)
Her-2 amplification	12 (18.5)
Basal-like	4 (6.2)
Pathological type	
Invasive cancer	51 (78.5)
Ductal carcinoma *in situ*	14 (21.5)
Size (cm)	
<5	48 (73.9)
≥5	17 (26.2)
Ki-67 (%)	
<14	12 (18.5)
≥14	53 (81.5)

CTC, circulating tumor cells; SD, standard deviation.

### Claudin-4

**FIGURE 1:**
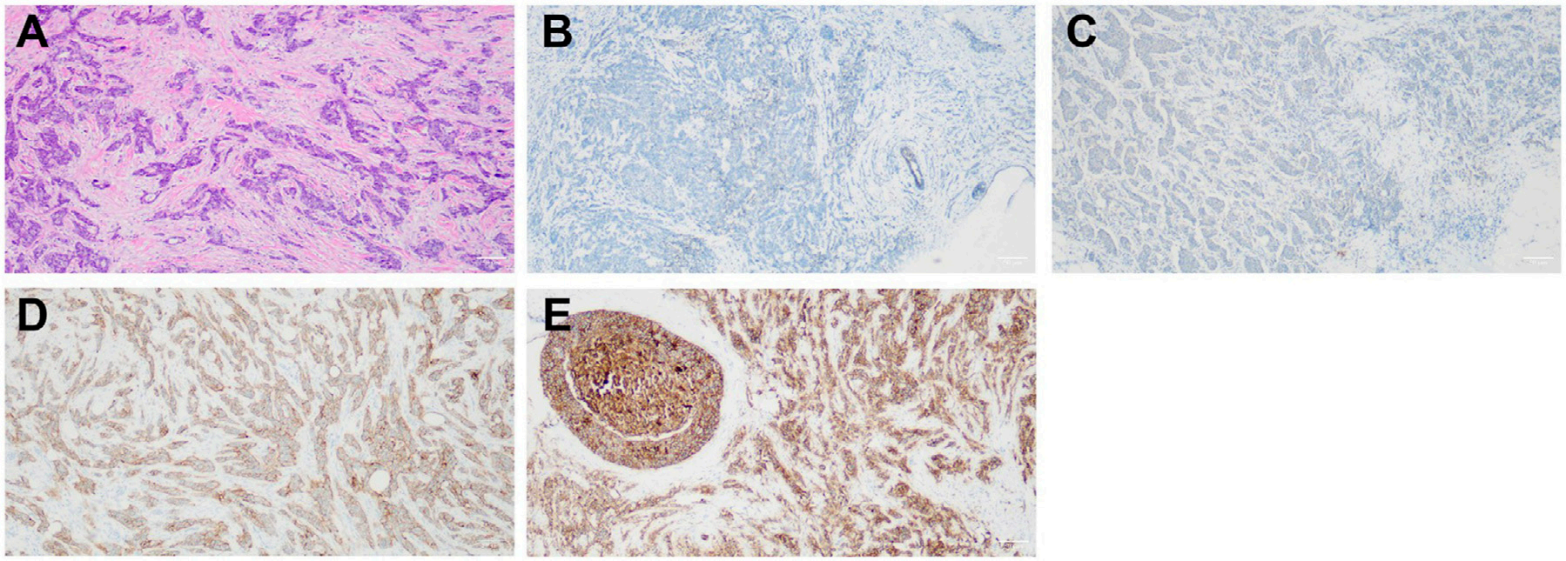
**(A)** Non-specific invasive carcinoma with HE staining (×100). **(B)** Non-specific invasive carcinoma with negative claudin-4 (SP ×100). **(C)** Non-specific invasive carcinoma claudin-4 (+, SP ×100). **(D)** Non-specific invasive carcinoma claudin-4 (2+, SP ×100). **(E)** Non-specific invasive carcinoma claudin-4 (3+, SP ×100).

Among the 65 patients, there were 11 Claudin-4-negative tumors, 27 with Claudin-4 +, 22 with Claudin-4 2+, and five with Claudin-4 3+ (Figure 1). Most patients with high Claudin-4 expression had lymph node metastasis (55.6% vs. 15.8%, *p* < 0.001 vs. without low Claudin-4 expression). Most patients with high Claudin-4 expression had a tumor >5 cm (51.9% vs. 7.9%, *p* < 0.001 vs. low Claudin-4 expression) (Table 2).

**TABLE 2:** Comparison of Claudin-4 in patients’ age, lymph node metastasis, and molecular classification.

Pathological parameters	Claudin-4 low *n* = 38	Claudin-4 high *n* = 27	*p*
Age (years)			
<50	19 (50.0)	15 (55.55)	0.659
≥50	19 (50.0)	12 (44.44)	
Lymph node metastasis			
No	32 (84.2)	12 (44.4)	<0.001
Yes	6 (15.8)	15 (55.6)	
Molecular			
Luminal A	3 (7.9)	4 (14.8)	0.760
Luminal B	25 (65.8)	17 (63.0)	
Her-2 amplification	8 (21.1)	4 (14.8)	
Basal-like	2 (5.3)	2 (7.4)	
Pathological type			
Invasive cancer	32 (84.2)	19 (70.4)	0.181
Ductal carcinoma *in situ*	6 (15.8)	8 (29.6)	
Size (cm)			
<5	35 (92.1)	13 (48.1)	<0.001
≥5	3 (7.9)	14 (51.9)	
Ki-67 (%)			
<14	6 (15.8)	6 (22.2)	0.510
≥14	32 (84.2)	21 (77.8)	

### Circulating tumor cells

CanPatrol CTC enrichment and *in situ* hybridization were used to monitor the number and classification of CTC in the blood of 65 patients. Red fluorescence was used to represent epithelial CTC, and green fluorescence was used to represent interstitial CTC. CTC was divided into epithelial type (E-CTC), mixed type (E/M-CTC), and interstitial type (M-CTC). The number of CTCs in patients aged ≥50 was significantly high (11.8 vs. 9.1, *p* < 0.001 vs. Age < 50). The number of CTCs in patients with lymph node metastasis was high (11.2 vs. 6.5, *p* < 0.001 vs. without lymph node metastasis). There are significant differences in the number of CTCs in different molecular types of breast cancer (*p* < 0.001) (Table 3).

**TABLE 3:** Relationship between CTCS and pathological parameters in patients with breast cancer.

Pathological parameters	Number of cases (%) *n* = 65	CTC count (*x* ± *s*)	*p*
Age (years)			
<50	34 (52.4)	9.08 ± 2.63	<0.001
≥50	31 (47.6)	11.77 ± 2.31	
Lymph node metastasis			
No	44 (67.7)	6.59 ± 2.28	<0.001
Yes	21 (32.3)	11.23 ± 1.54	
Molecular			
Luminal A	7 (10.7)	10.14 ± 2.11	<0.001
Luminal B	42 (64.6)	11.33 ± 2.56	
Her-2 amplification	12 (18.5)	8.58 ± 2.07	
Basal-like	4 (6.1)	6.08 ± 1.82	
Pathological type			
Invasive cancer	51 (78.5)	11.8 ± 2.82	0.334
Ductal carcinoma *in situ*	14 (21.5)	10.71 ± 2.52	
Size (cm)			
<5	48 (73.8)	11.41 ± 2.92	0.755
≥5	17 (26.2)	11.10 ± 2.18	
Ki-67 (%)			
<14	12 (18.5)	11.67 ± 2.49	0.694
≥14	53 (81.5)	11.31 ± 2.89	

### Circulating tumor cells and Claudin-4

**FIGURE 2:**
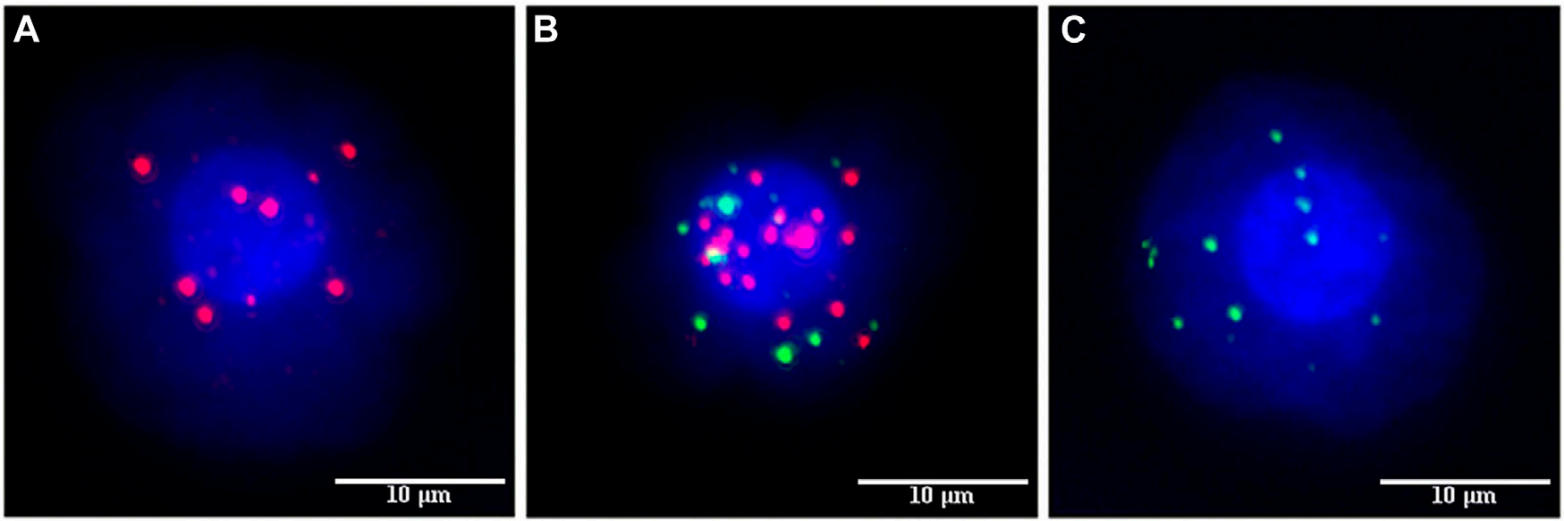
Fluorescence marking different types of circulating tumor cells (CTCs). **(A)** EPCAM, CK8, and cK8/18 epithelial fluorescent markers emit red fluorescence to label epithelial circulating tumor cells. **(B)** Hybrid circulating tumor cells were labeled with both red and green fluorescence. **(C)** Vimentin mesenchymal fluorescent marker emits green fluorescence to label mesenchymal circulating tumor cells.

**FIGURE 3:**
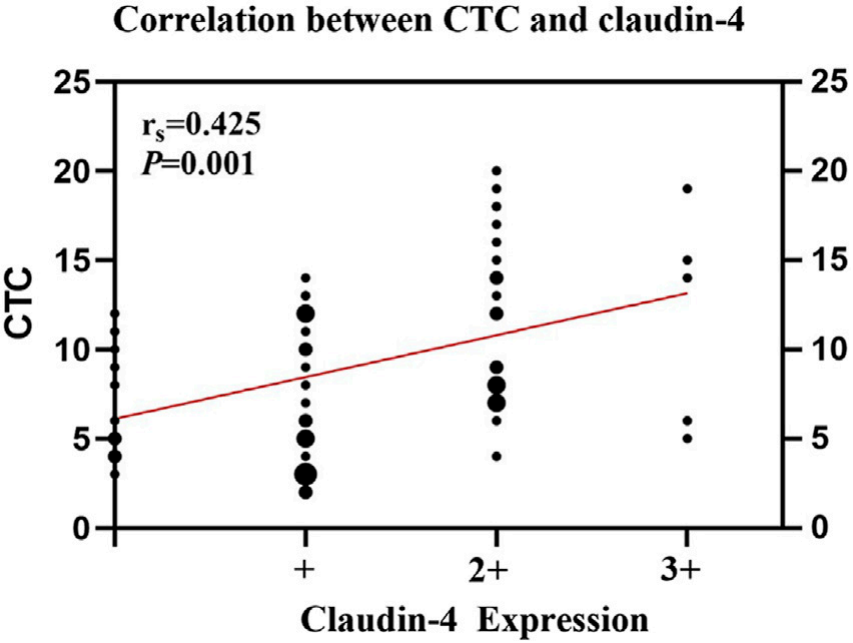
Spearman rank correlation analysis between circulating tumor cells (CTCs) and Claudin-4 expression.

The number of CTCs was higher in patients with high Claudin-4 expression (11.7 vs. 7.4, *p* < 0.001). A more detailed analysis revealed that a high Claudin-4 expression was associated with a lower count of E-CTCs (3.4 vs. 5.0, *p* = 0.033) but high counts of E/M-CTCs (5.0 vs. 3.5, *p* = 0.021) and M-CTCs (4.4 vs. 1.1, *p* < 0.001) (Table 4 and Figure 2). Spearman correlation analysis showed that the intensity of Claudin-4 was positively correlated with CTC (r_s_ = 0.43, *p* = 0.001) (Figure 3).

**TABLE 4:** Comparison of Claudin-4 in patient CTC types.

CTC types (/mL)	Claudin-4 low (*n* = 38)	Claudin-4 high (*n* = 27)	*p*
Total CTC	7.42 ± 2.23	11.70 ± 2.35	<0.001
E-CTC	4.97 ± 3.04	3.41 ± 2.57	0.033
E/M-CTC	3.50 ± 2.83	4.04 ± 2.17	0.021
M-CTC	1.12 ± 1.94	4.41 ± 2.11	<0.001

CTC, circulating tumor cells; E, epithelial; M, mesenchymal.

### Correlation analysis between CTC classification and lymph node metastasis

E-CTC in breast cancer patients with lymph node metastasis was significantly higher than that in the lymph node metastasis group (3.4 vs. 1.4, *p* < 0.001). E/M-CTC was significantly lower than that in the lymph node metastasis group (1.5 vs. 4.3, *p* = 0.002), and the M-CTC count was lower than that in the lymph node metastasis group (1.6 vs. 6.4, *p* < 0.001) (Table 5).

**TABLE 5:** The relationship between the type of CTC and Lymph node metastasis.

CTC types (/mL)	No lymph node metastasis (*n* = 44)	Lymph node metastasis (*n* = 21)	*p*
E-CTC	3.37 ± 1.67	1.41 ± 1.01	<0.001
E/M-CTC	1.58 ± 1.40	4.33 ± 1.71	0.002
M-CTC	1.62 ± 2.61	6.45 ± 2.44	<0.001

### Survival analysis of Claudin-4 expression level cancer

**FIGURE 4:**
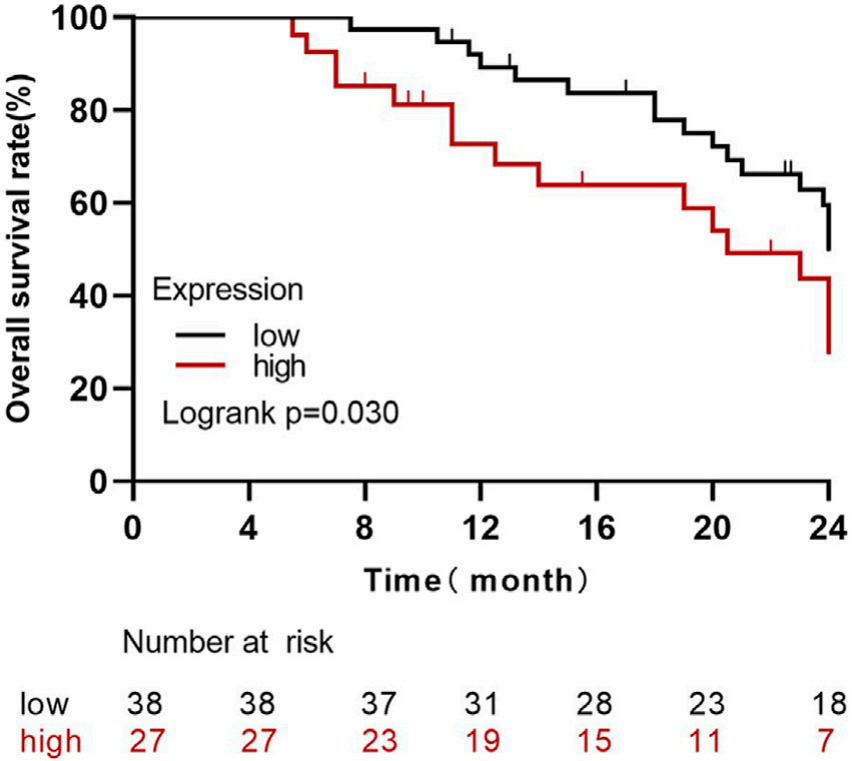
Survival analysis of Claudin-4 expression level and prognosis of breast cancer.

By the last follow-up, the average survival time of 65 patients with high expressions of Claudin-4 (*n* = 27) was 18.22 months, and the average survival time of the low-expression group (*n* = 38) was 21.81 months. The survival rate of the high-expression group was significantly lower than that of the low-expression group (Log Rank Χ^2^ = 4.71, *p* = 0.030) (Figure 4).

### A multivariate COX regression analysis model was constructed

The results showed that CTC counts (HR = 1.3, *p* < 0.001), Claudin-4 (HR = 4.6, *p* = 0.008), and lymph node metastasis (HR = 12.9, *p* = 0.001) were independent prognostic factors for poor prognosis (Table 6).

**TABLE 6:** Multivariate COX regression risk model.

Factors	*B*	SE	Wald	*P*	HR (95% CI)
Total CTC	0.25	0.06	18.13	<0.001	1.29 (1.147–1.450)
Claudin-4 (low vs. high)	1.53	0.57	7.14	0.008	4.6 (1.504–14.248)
Lymphatic metastasis (-vs +)	2.55	0.49	27.44	0.001	12.86 (4.945–33.431)

### The COX risk regression model of CTC classification and prognosis of breast cancer patients was constructed

Our analysis showed that E/M-CTC (HR = 1.9, *p* = 0.001) and M-CTC (HR = 1.5, *p* = 0.001) were independent influencing factors of poor prognosis in breast cancer patients (Table 7).

**TABLE 7:** Multivariate COX regression model of CTC typing.

Factors	*B*	SE	Wald	*P*	HR (95% CI)
E-CTC	0.19	0.24	0.71	0.400	1.22 (0.768–1.936)
E/M-CTC	0.66	0.19	11.46	0.001	1.93 (1.319–2.827)
M-CTC	0.40	0.12	11.62	0.001	1.49 (1.186–1.883)

## Discussion

Breast cancer with metastases can reduce survival rates, so markers for early diagnosis and prognostic monitoring are urgently needed for breast cancer patients. Based on this study, we can explore the prognosis of patients by assessing the expression of Claudin-4 and circulating tumor cells in peripheral blood.

The results showed that the CTC count and classification in breast cancer are associated with the expression of Claudin-4. The results may help define novel molecular targets for the early diagnosis and prognosis of breast cancer. Future studies should examine the diagnostic and prognostic values of CTCs and Claudin-4 in breast cancer patients.

Claudins are the most important structural and functional component of tight junction transmembrane proteins and can maintain cell-to-cell molecular flow and cell polarity [24]. Claudins are overexpressed or silenced in pancreatic cancer, colon cancer, ovarian cancer, breast cancer, and other solid tumors [25]. In particular, Claudin-4 can directly or indirectly promote tumor metastasis through the second extracellular loop structure [26]. The changes in the expression of Claudin-4 can modify the structure of the tight junctions and adhesion between cells, leading to tumor metastasis and spread [27]. Claudin-4 is overexpressed in ovarian and breast cancers [28]. Kolokytha et al. [29] showed that the positive expression of Claudin-4 in triple-negative breast cancer might be a marker of good prognosis. Radi et al. [30] showed that Claudin-4 was related to the expression of D240 (a lymphatic vessel marker) in prostate cancer, and Claudin-4 is associated with lymph node metastasis and a marker of poor prognosis. Moreover, the present study also confirmed that the expression level of Claudin-4 was associated with lymph node metastasis and tumors ≥5 cm, suggesting that higher Claudin-4 expression may be associated with a poor prognosis in breast cancer. In our study, Claudin-4 was poorly significantly associated with molecular subtypes of breast cancer, and Sara Ricardo’s study showed that a single IHC for Claudin-4 is not sufficient to assess and identify molecular subtypes of breast cancer and that additional markers, such as CSC (cancer stem cell) markers, are needed to improve subgroup identification [31]. The survival time of the Claudin-4 high-expression group was significantly lower than that of the low-expression group, and a multivariate COX regression model was established to find that high expression of Claudin-4 was an independent factor affecting the poor prognosis of breast cancer.

The present study showed that the number of E/M-CTC and M-CTC in the lymph node metastasis group was higher than that in the lymph node non-metastasis group and the numbers of CTCs in the high Claudin-4 group were higher than in the low expression level group, mainly due to the E/M- and M-CTCs since the E-CTCs were lower in the high Claudin-4 group. These results suggest that Claudin-4 is involved in EMT and tumor metastasis and recurrence. The Spearman correlation analysis also showed that CTCs in peripheral blood were positively correlated with the expression level of Claudin-4. The Multivariate COX regression model showed that Claudin-4, lymph node metastasis, and CTC classification E/M-CTC and M-CTC were adverse factors for the prognosis of breast cancer patients. Therefore, both Claudin-4 and CTC might be used to evaluate the prognosis and recurrence risk of patients with breast cancer. Meanwhile, the high expression of Claudin-4 might regulate or be regulated by the tumor EMT process, but the present study could not determine cause-to-effect relationships.

This study has limitations. It was a single-center study with a small sample size. Only CTCs and Claudin-4 were examined. Future studies should look at the correlations of multiple biomarkers and in-depth mechanism research to help personalize the prognosis of breast cancer. It was a cross-sectional study, and cause-to-effect relationships could not be determined.

In conclusion, CTC count and classification in breast cancer are associated with the expression of Claudin-4. CTC count and classification and the expression level of Claudin-4 may be used for the early diagnosis and prognosis of breast cancer. Future large studies should attempt to investigate the diagnostic and prognostic value of CTCs and claudin-4 in breast cancer patients.

## Data Availability

The original contributions presented in the study are included in the article/supplementary material, further inquiries can be directed to the corresponding author.
